# Refractory Invasive Pulmonary Aspergillosis Following Treatment of Acute Rejection in a Kidney Transplant Recipient: A Case Report

**DOI:** 10.1155/crit/8425439

**Published:** 2026-09-30

**Authors:** Georges Aboukasm, Mariella Ortigosa-Goggins, Jacques Simkins

**Affiliations:** ^1^ University of Miami Miller School of Medicine Miami Florida USA miami.edu; ^2^ Miami Transplant Institute Jackson Memorial Hospital Miami Florida USA jhsmiami.org; ^3^ Division of Nephrology University of Miami Miller School of Medicine Miami Florida USA miami.edu; ^4^ Division of Infectious Disease University of Miami Miller School of Medicine Miami Florida USA miami.edu

**Keywords:** acute rejection, antifungal therapy, *Aspergillus terreus*, invasive pulmonary aspergillosis, kidney transplantation, triple antifungal therapy

## Abstract

Invasive pulmonary aspergillosis (IPA) is an uncommon but highly morbid complication after solid organ transplantation, particularly following treatment for acute rejection. We report a case of refractory IPA caused by *Aspergillus terreus* in a 70‐year‐old kidney transplant recipient, diagnosed 2 weeks after receiving thymoglobulin and pulse corticosteroids for acute T cell–mediated rejection. The patient presented with acute hypoxic respiratory failure requiring intubation, and a chest computed tomography scan demonstrated a large, necrotic, mass‐like cavitary opacity in the left upper lobe and lingula. Serum and bronchoalveolar lavage (BAL) galactomannan (GM) were markedly elevated, the *Aspergillus* PCR was positive, and BAL culture grew *A. terreus*. Initial treatment included voriconazole and micafungin, and immunosuppression was substantially reduced. Although the patient initially improved radiographically, his outpatient course was complicated by persistently high GM antigenemia despite antifungal therapy, requiring the addition of terbinafine as a third antifungal agent. Serum GM eventually became negative after 33 weeks of treatment, and adjunctive micafungin and terbinafine were discontinued at Week 42, with the continuation of a long‐term azole. This case highlights the risk of rapidly progressive IPA following rejection therapy in kidney transplant recipients and underscores the importance of prompt species identification, serial GM and radiographic monitoring, and timely adjustment of both antifungal therapy and immunosuppression.

## 1. Introduction

Invasive pulmonary aspergillosis (IPA) is an uncommon but serious complication among kidney transplant recipients (KTRs), with a reported incidence ranging from 0.2% to 14%, although this incidence is generally lower than that reported in other solid organ transplant (SOT) recipients [1, 2]. However, because kidney transplantation is performed more frequently than other SOTs, the absolute burden of IPA in this population remains clinically important. IPA is associated with high mortality in KTRs, with reported rates of approximately 40%–60%, underscoring the importance of early diagnosis and prompt initiation of antifungal therapy [1–3].

Risk factors for IPA in KTRs include advanced age, diabetes mellitus, delayed graft function (DGF), acute graft rejection, and intensified immunosuppression, particularly antirejection therapy [1, 4, 5]. Management often requires prolonged antifungal treatment, therapeutic drug monitoring (TDM), and a reduction of immunosuppression when feasible, creating a difficult balance between infection control and allograft preservation. We present a case of refractory IPA due to *Aspergillus terreus* after treatment of acute rejection in a KTR, requiring upfront reduction of immunosuppression, triple antifungal therapy, and close TDM.

## 2. Case Presentation

A 70‐year‐old male patient with end‐stage kidney disease secondary to diabetic nephropathy underwent a preemptive deceased donor kidney transplantation. Induction immunosuppression consisted of rabbit anti‐thymocyte globulin 2.5 mg/kg, basiliximab (two doses of 20 mg), and methylprednisolone (three doses of 500 mg IV). Maintenance immunosuppression consisted of tacrolimus (2 mg PO BID) for 2 weeks followed by belatacept protocol (5 mg/kg every two weeks) and back to tacrolimus 2 mg PO BID, mycophenolate mofetil (1 g PO BID) and prednisone (20 mg PO daily). His immediate posttransplant course was complicated by DGF, requiring hemodialysis. Seven weeks posttransplant, a kidney transplant biopsy performed for persistent graft dysfunction demonstrated acute T cell–mediated rejection Banff grade IA. He was treated with rabbit anti‐thymocyte globulin 2 mg/kg and pulse corticosteroids (total dose was 4.5 mg/kg of rabbit anti‐thymocyte globulin, counting induction therapy), followed by a steroid taper (60–5‐mg taper).

Nine weeks posttransplant (2 weeks following rejection therapy), he was admitted to the hospital with severe volume overload and anasarca (20 kg above dry weight) and was treated with hemodialysis and intravenous diuresis. However, shortly after admission he developed acute hypoxic respiratory failure requiring intubation and transfer to the intensive care unit (ICU). A chest computed tomography (CT) scan revealed a large 13.8 × 8.3 × 14.0 − cm mass‐like opacity with central hypodensity in the left upper lobe and lingula, concerning for necrotizing pneumonia versus malignancy (Figure 1).

**Figure 1:**
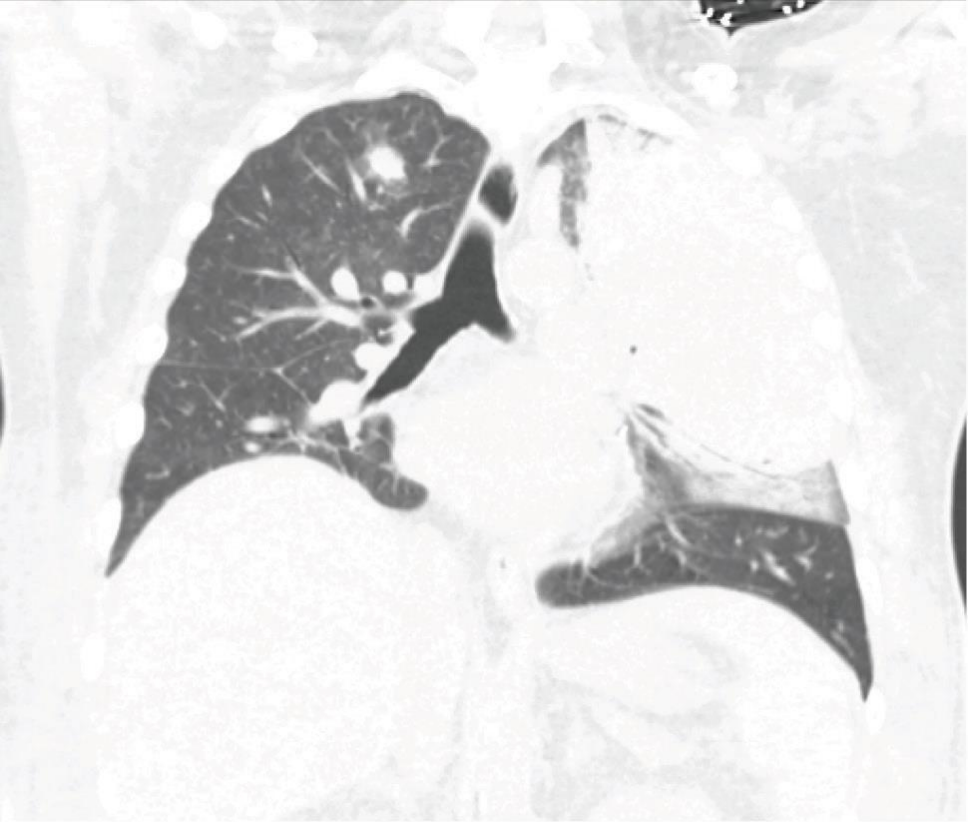
Computed tomography of chest (coronal view). A 13.8 × 8.3 × 14.0 − cm mass‐like opacities noted in the left upper lobe and superior segment of the lingula with a central hypodensity.

Microbiologic and serologic evaluation strongly supported IPA. Serum *Aspergillus* galactomannan (GM) was markedly elevated at > 7.44 (Figure 2), and serum *β*‐D‐glucan was > 500 pg/mL. Bronchoalveolar lavage (BAL) *Aspergillus* GM was > 6.5, and the *Aspergillus* PCR was positive for *Aspergillus fumigatus* and *A. terreus*, whereas the mycology BAL culture grew *A. terreus*. Blood cultures were negative. Broad‐spectrum antibacterial therapy was used early in the ICU course empirically for possible superimposed bacterial pneumonia.

**Figure 2:**
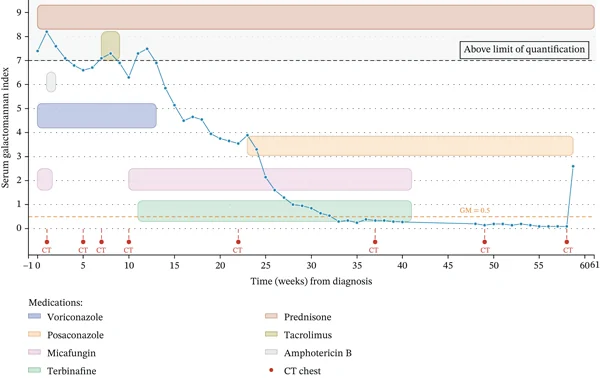
Tracking of serum galactomannan (GM), medications, and computed tomography scans throughout clinical course. Serum GM was tracked to assess clinical improvement. A serum GM > 7 was above limit of quantification, and serum GM < 0.5 was considered negative. Medications throughout the course of treatment can be found in the colored boxes with each color corresponding to a different medication noted at the top of the graph. Computed tomography scans are listed at the bottom corresponding with the time they were obtained.

Voriconazole was initiated on Day 0 of suspected IPA (loading dose: 6 mg/kg IV every 12 h for two doses, followed by 4 mg/kg every 12 h). Micafungin 150 mg IV daily was added on Day 1. Liposomal amphotericin B 500 mg IV daily was administered briefly from Days 5 to 6 but was discontinued upon microbiological confirmation of *A. terreus* due to its intrinsic resistance. Antifungal susceptibility testing performed at a reference laboratory subsequently showed low minimum inhibitory concentrations (MIC) to posaconazole (MIC ≤ 0.03 mcg/mL), voriconazole (MIC 0.25 mcg/mL), and isavuconazole (MIC 0.25 mcg/mL).

Immunosuppression was reduced in the setting of severe IPA. Mycophenolate mofetil and tacrolimus were held, and prednisone 5 mg daily was continued. The thoracic surgical team evaluated him for a possible lobectomy, but operative intervention was deferred. Diagnostic nasal endoscopy and sinus CT showed no evidence of invasive fungal sinusitis. Brain magnetic resonance imaging demonstrated acute embolic infarcts deemed atypical for fungal central nervous system involvement, and an ophthalmologic evaluation for left eye redness noted possible vitritis, prompting vitreous aspiration and the administration of intravitreal vancomycin and ceftazidime, though cultures were nondiagnostic for ocular aspergillosis. A left pneumothorax developed 2 weeks into the ICU admission and required chest tube placement. Donor information was reviewed, and donor‐derived aspergillosis was excluded.

Voriconazole was switched from 450 mg IV every 12 h to 550 mg PO every 12 h, after a voriconazole trough of 1.7 *μ*g/mL to target a higher level. One week later, he developed suspected voriconazole‐related thrombocytopenia coinciding with a supratherapeutic voriconazole level of 5.4 *μ*g/mL, necessitating a dose reduction back to voriconazole 450 mg every 12 h, with subsequent stabilization of platelets around 52,000/*μ*L. A follow‐up chest CT at 1 month demonstrated radiographic improvement, with a decreased size of the left lung mass‐like cavitary opacity, a decreased right cavitary nodule, and improvement in widespread ground glass opacities and clustered nodular densities. Micafungin was subsequently stopped, tacrolimus (0.5 mg PO BID) was resumed, prednisone was increased from 5 to 10 mg daily, and the patient was discharged to acute inpatient rehabilitation after a 6‐week admission.

Despite initial improvement, the outpatient course was marked by refractory fungal disease. By Week 11 postdiagnosis, serum *Aspergillus* GM remained markedly elevated (> 7.30), accompanied by a worsening cough, with a follow‐up CT at Week 10 scan showing the dominant cavitary lesion slightly decreased in size compared with previous scans. Suspecting that the immunosuppression resumption contributed to impaired fungal clearance, tacrolimus was discontinued permanently, prednisone was reduced to 5 mg daily, and micafungin 150 mg daily was restarted. Terbinafine 500 mg every 12 h was additionally added at this time as a third antifungal therapy for refractory disease. At Week 12, serum GM began to decline from > 7.53, which was the last time it was above the limit of quantification (Figure 2). Due to persistently low voriconazole levels (partially attributed to concurrent oral calcium acetate use, which was held), therapy was switched to delayed‐release posaconazole (loading dose: 300 mg PO every 12 h for two doses, followed by 300 mg daily) at Week 23. Subtherapeutic posaconazole levels ultimately required dose escalation up to 400 mg every 12 h of posaconazole to achieve target levels (> 1.5 mg/L). Serial CT imaging over the following months demonstrated gradual improvement of the dominant left upper lobe cavitary lesion and surrounding inflammation, though residual cavitation and nodules persisted. Serum GM became negative for the first time at Week 33 (Figure 2) into diagnosis. At Week 42 postdiagnosis, adjunctive micafungin and terbinafine therapy were stopped, and he was kept on long‐term posaconazole. Therapeutic drug levels and serial laboratory values are depicted in Figure 3.

**Figure 3:**
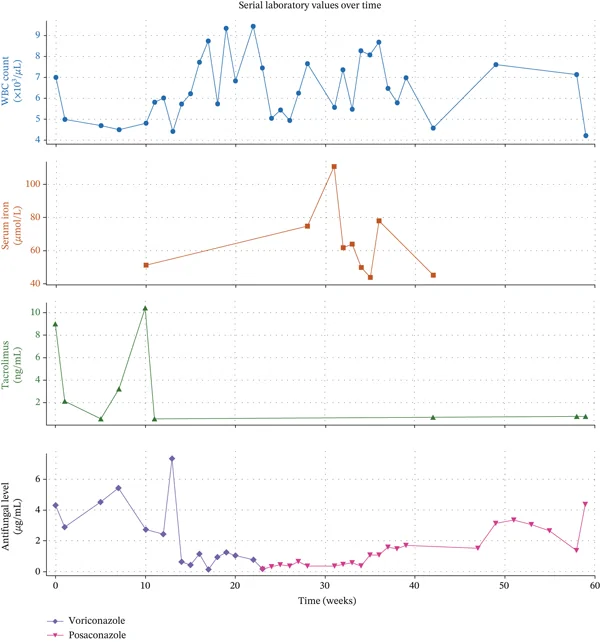
Serial laboratory values over time since initial diagnosis. White blood cell (WBC) count, serum iron, tacrolimus levels, and antifungal levels (both voriconazole and posaconazole) are depicted over the course of initial aspergillosis diagnosis. Therapeutic levels for tacrolimus are 3–12 ng/mL. Therapeutic levels for voriconazole and posaconazole are 1–5.5 *μ*g/mL and 1–3.75 *μ*g/mL, respectively.

Approximately 13 months after his initial presentation, the patient was readmitted from rehabilitation following an acute decline in mental status. Chest CT showed worsening multifocal bilateral airspace disease, consolidation around the prior left upper lobe cavity, and new bilateral loculated pleural effusions. He developed progressive respiratory failure requiring mechanical ventilation. A BAL viral panel returned positive for rhinovirus; however, BAL and blood cultures were negative. Serum *Aspergillus* GM remained negative; however, a subsequent BAL GM was positive (> 2.6). Despite treatment with broad‐spectrum antibacterials, posaconazole, and micafungin, the patient developed progressive respiratory failure, poor neurologic status, profound deconditioning, and a low likelihood of meaningful recovery. Following goals‐of‐care discussions, the family elected comfort measures, and he died later that week.

## 3. Discussion

This case highlights the potential for severe IPA to develop rapidly after treatment for rejection in KTRs. Although our patient had several established risk factors for posttransplant IPA, including diabetes, posttransplant hemodialysis due to DGF, and acute graft rejection, [6] IPA developed 2 weeks after rejection therapy, which included rabbit anti‐thymocyte globulin (total dose was 4.5 mg/kg, counting induction therapy), suggesting that intensified immunosuppression may have been a key precipitating factor. A study at the University of Wisoconsin found that anti‐thymocyte globulin was independently associated with increased risk of invasive fungal infections (adjusted HR 1.52) [7]. Similarly, a retrospective case‐control study from South Korea found that approximately two‐thirds of invasive fungal infections after kidney transplantation occurred following rejection therapy, whereas Heylen et al. [3] reported IPA in 0.9% of 6,146 KTRs, increasing to 5.7% among those who received ≥1 g of methylprednisolone [8]. The period immediately following pulse corticosteroids or lymphocyte‐depleting antirejection therapy should therefore be considered a high‐risk window for rapidly progressive fungal disease. New fever or pulmonary symptoms in this setting should prompt early CT imaging, fungal biomarker testing, and bronchoscopy when feasible [9]. However, in critically ill patients, as in our case, empiric antifungal therapy may need to be initiated before bronchoscopic confirmation [9].

Although the 2019 American Society of Transplantation Infectious Diseases Community of Practice guidelines do not recommend routine combination therapy for IPA, combination treatment may be considered in fulminant or disseminated infection [10]; similar to our patient, who ultimately required triple antifungal therapy, an approach that has been described only rarely in SOT recipients with refractory or fulminant IPA. In two prior reports, patients developed *A. fumigatus* infection and were treated with a combination of amphotericin B, caspofungin, and voriconazole, although one case occurred in a heart transplant patient and the other after multivisceral transplantation [11, 12]. To our knowledge, this is the first reported case of early‐onset kidney transplant‐associated IPA following rejection therapy managed with triple antifungal therapy, specifically an azole, terbinafine, and micafungin. Studies showing the concurrent use of terbinafine and azole are limited; however, multiple in vitro studies have demonstrated synergistic or additive interactions between terbinafine and azoles against *Aspergillus* species, including *A. terreus* specifically [13, 14]. Interestingly, limited activity of terbinafine was observed against *A. fumigatus* [15]. Furthermore, a pilot randomized study of patients with chronic IPA showed complete or partial response to terbinafine in 67% of patients [16]. Additionally, early species‐level identification was particularly important in this case because *A. terreus* has clinically relevant differences in antifungal susceptibility compared with other more common *Aspergillus* species. A large retrospective study found that *A. terreus* was associated with a significantly lower response rate (21% vs. 38%) and higher IPA‐associated mortality (51% vs. 30%) compared with non‐*A. terreus* species, even after adjusting for the antifungal regimen [17]. Once *A. terreus* is identified, amphotericin B becomes a less reliable option because of its reduced activity against this species and its potential nephrotoxicity, whereas voriconazole‐based therapy is generally preferred [9, 18–20].

Although voriconazole remains the preferred first‐line therapy for invasive aspergillosis, isavuconazole and posaconazole have also been shown to be noninferior in prospective randomized trials, providing additional options when voriconazole cannot be used or therapeutic levels cannot be achieved [21–24]. TDM was central to management in this case and is recommended after steady state is achieved for patients receiving triazole‐based therapy [9, 25]. Voriconazole exposure was difficult to optimize in our patient, with variable levels and subsequent persistently subtherapeutic troughs despite dose adjustments. This was potentially due to drug interactions, impaired absorption, or *CYP2C19* pharmacogenomic variability, although *CYP2C19* testing was not performed in our patient. Given the inability to achieve therapeutic voriconazole levels, therapy was transitioned to posaconazole.

In our patient, the failure to achieve a ≥ 35% decline in serum GM by Day 7, together with persistent cavitary disease, supported intensification to triple antifungal therapy and the continuation of treatment until antigenemia and radiologic progression had clearly improved. Persistent or rising serum GM antigenemia, defined as an optical density index ≥ 2.0, an increase ≥ 0.25 from baseline, or a failure to decline ≥ 35% in the first week, and/or cavitary lung disease argue strongly against early de‐escalation of therapy [26]. In SOT recipients, the Infectious Diseases Society of America recommends a minimum 12‐week course, with treatment failure defined after ≥ 15 days when the GM remains ≥ 2.0 or lesions enlarge > 25% despite stable immunity [9]. Such failure mandates salvage strategies: switching the antifungal class, adding combination therapy, tapering immunosuppression when feasible, and considering surgical resection of necrotic foci. Persistent antigenemia portends a 4‐ to 6‐fold rise in mortality [27]. Furthermore, as shown in Brodoefel′s series, radiographic resolution often lags behind clinical resolution and when serum GM becomes negative, with a median of 80 days to radiologic cure, and the presence of cavitation delaying resolution 2.5‐fold [28]. Therefore, therapy should continue until both antigen levels and imaging clearly improve, as was done in our patient.

In conclusion, this case describes the use of triple antifungal therapy for IPA in a KTR following acute rejection therapy with lymphocyte depleting agent. It emphasizes the importance of early species‐level identification, prompt initiation of azole‐based therapy, TDM, and close assessment of clinical, radiographic, and biomarker responses to guide therapy escalation when needed. Finally, infection control may require a substantial reduction or temporary withdrawal of immunosuppression, even at the risk of allograft injury or loss. Although this trade‐off is difficult, this case highlights that in life‐threatening IPA, patient survival must take precedence over graft preservation.

## Funding

No funding was received for this manuscript.

## Consent

No written consent has been obtained from the patients as there is no patient identifiable data included in this case report.

## Conflicts of Interest

The authors declare no conflicts of interest.

## Data Availability

Data sharing is not applicable to this article as no datasets were generated or analyzed during the current study.
